# Supplementary material for: SAGA1 and SAGA2 promote starch formation around proto-pyrenoids in Arabidopsis chloroplasts
Source: Proc Natl Acad Sci U S A. 2024 Jan 19;121(4):e2311013121. doi: 10.1073/pnas.2311013121 (PMC10823261; doi:10.1073/pnas.2311013121)
Supplement: Supplementary file 1 — Appendix 01 (PDF) [file pnas.2311013121.sapp.pdf]

PNAS

Supplementary Figures and Tables for

SAGA1 and SAGA2 promote starch formation around proto-pyrenoids in Arabidopsis chloroplasts

Nicky Atkinson[1,2], Rhea Stringer[3], Stephen R Mitchell[1], David Seung*[3], Alistair J. McCormick*[1,2]

[1]Institute of Molecular Plant Sciences, School of Biological Sciences, University of Edinburgh, EH9 3BF, UK
[2]Centre of Engineering Biology, University of Edinburgh, EH9 3BF, UK
[3]John Innes Centre, Norwich Research Park, NR4 7UH, UK

*Alistair J. McCormick

*David Seung

Email: alistair.mccormick@ed.ac.uk; david.seung@jic.ac.uk

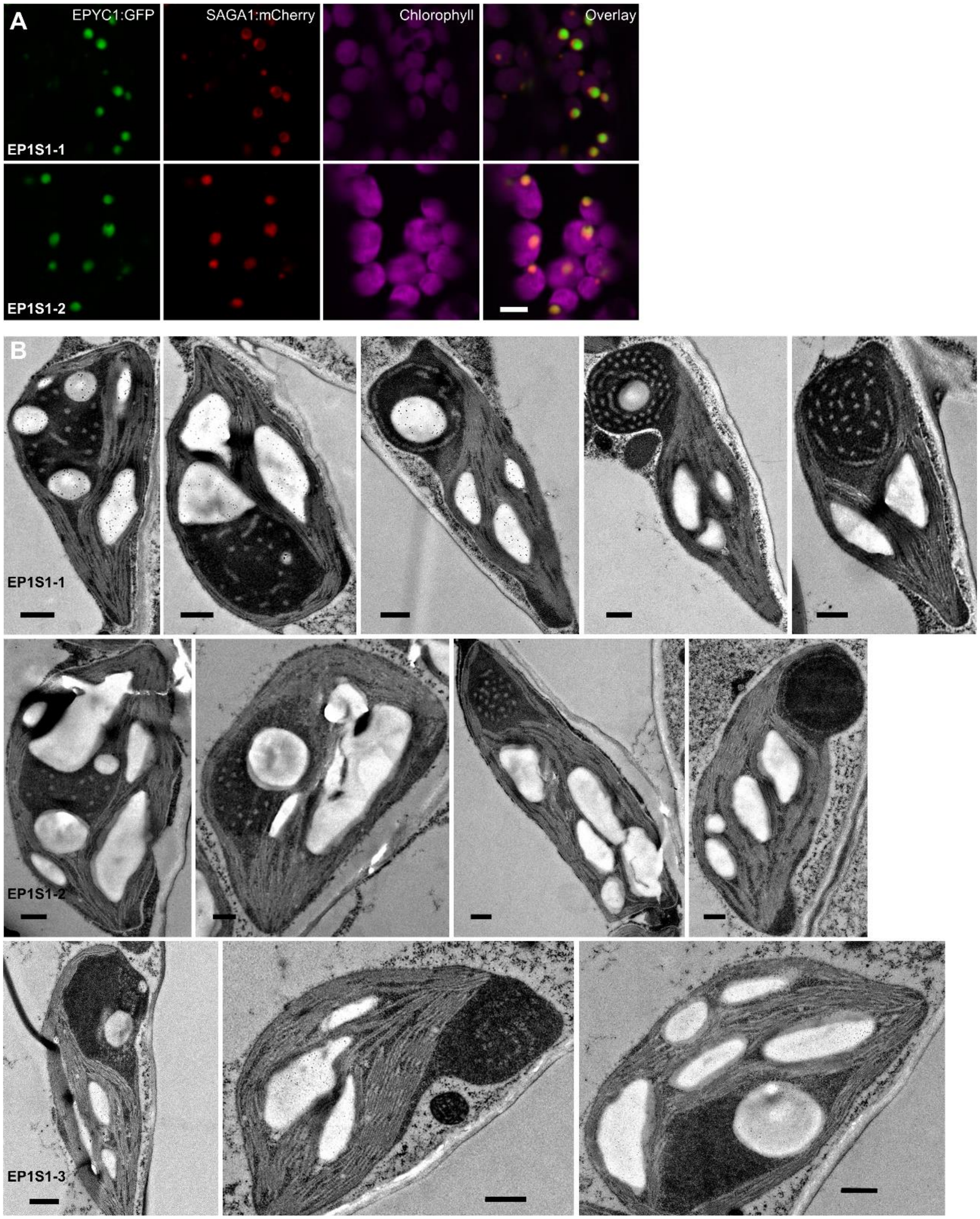

Figure S1. Additional confocal and TEM images for EP1S1 lines. A. Confocal image examples of alternative expression patterns for SAGA1::mCherry localised to the proto-pyrenoid from lines EP1S1-1 and EP1S1-2 (**Fig. 1*D***). Scale bar = 0.5 µm. **B.** Representative TEM image examples for EP1S1-1, EP1S1-2 and EP1S1-3. Scale bar = 0.5 µm.

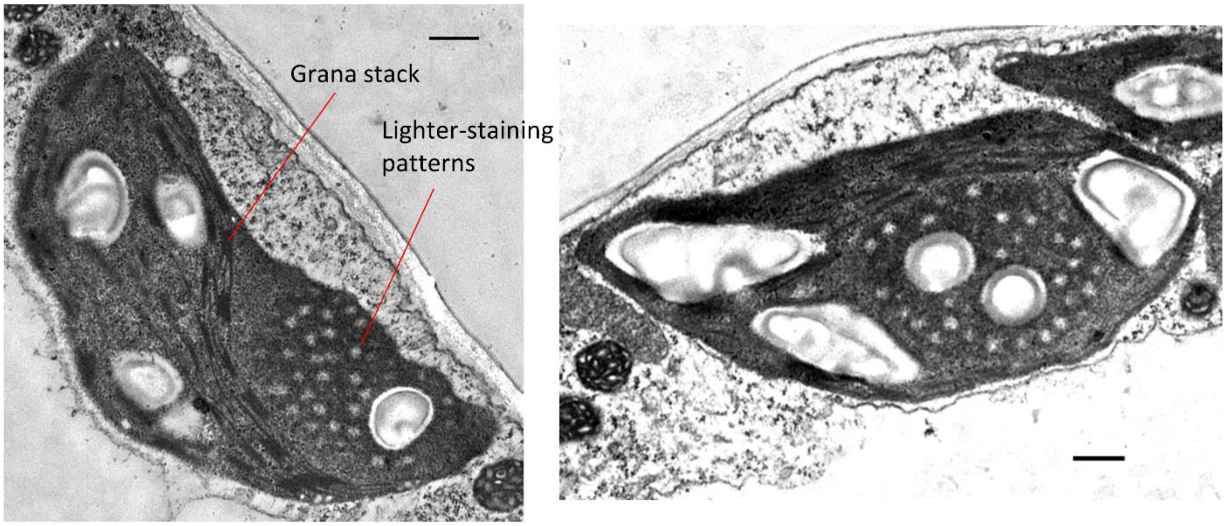

Figure S2. Lighter-staining regions in EP1S1-1 are not stained by osmication. Following fixation EP1S1-1 leaf samples were stained with 1% (w/v) osmium tetroxide in 0.05 M sodium cacodylate for 45 minutes. Samples were washed three times with phosphate buffered saline, before being dehydrated and embedded as normal. Membranes such as the visible grana stacks were stained black during this process, whilst the patterned structures in the condensate matrix remained lighter-coloured. Scale bar = 0.5 µm.

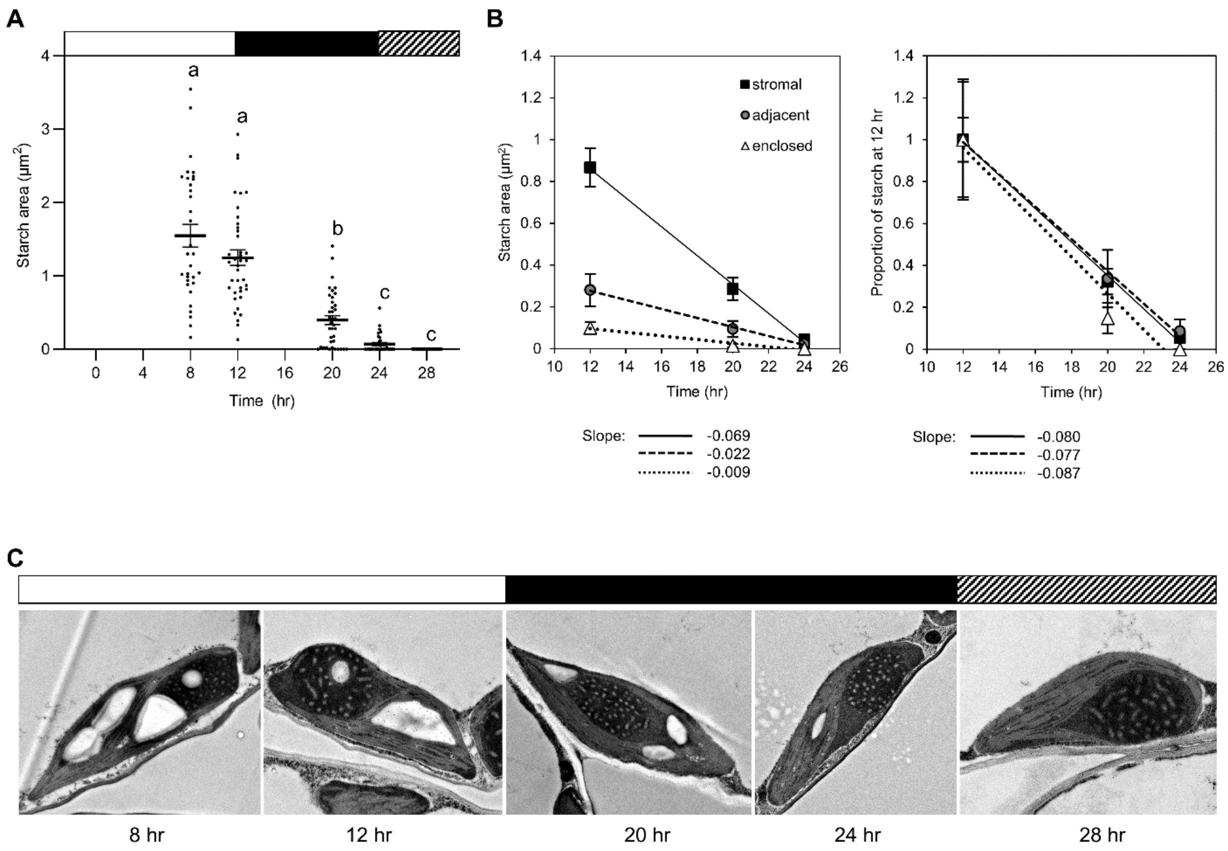

Figure S3. Total starch area and representative images in EP1S1-1 over time. A. Average of total areas for stroma, adjacent and enclosed starch granules in EP1S1-1 plants over time from **Fig. 2*G***. The averages at each time point represents the mean ± SEM of 22-39 images. Letters indicate significant difference ($p < 0.05$) as determined by one-way ANOVA followed by a Kruskal-Wallis post-hoc test. **B.** Starch degradation rates plotted for each starch type showing total starch area (left) and proportional starch area (right). Trendlines are fitted to the data and the values for the slopes given under each graph. **C.** Representative TEM images of starch abundance over time.

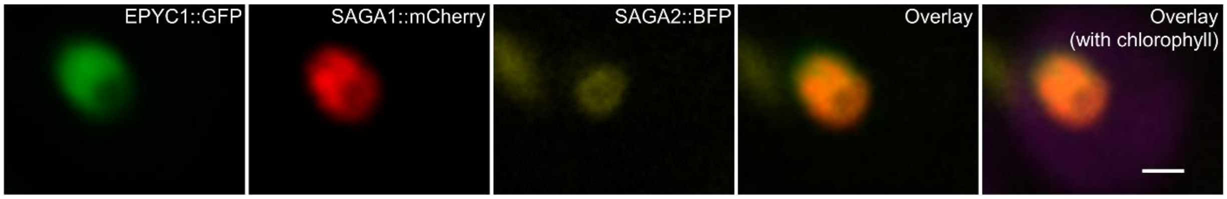

Figure S4. Representative confocal images of plants expressing EPYC1, SAGA1 and SAGA2 (EP1S1S2) with each transgene tagged with a unique fluorophore. Scale bar = 1 µm.

EP1S1S2-1

EP1S1S2-2

EP1S1S2-3

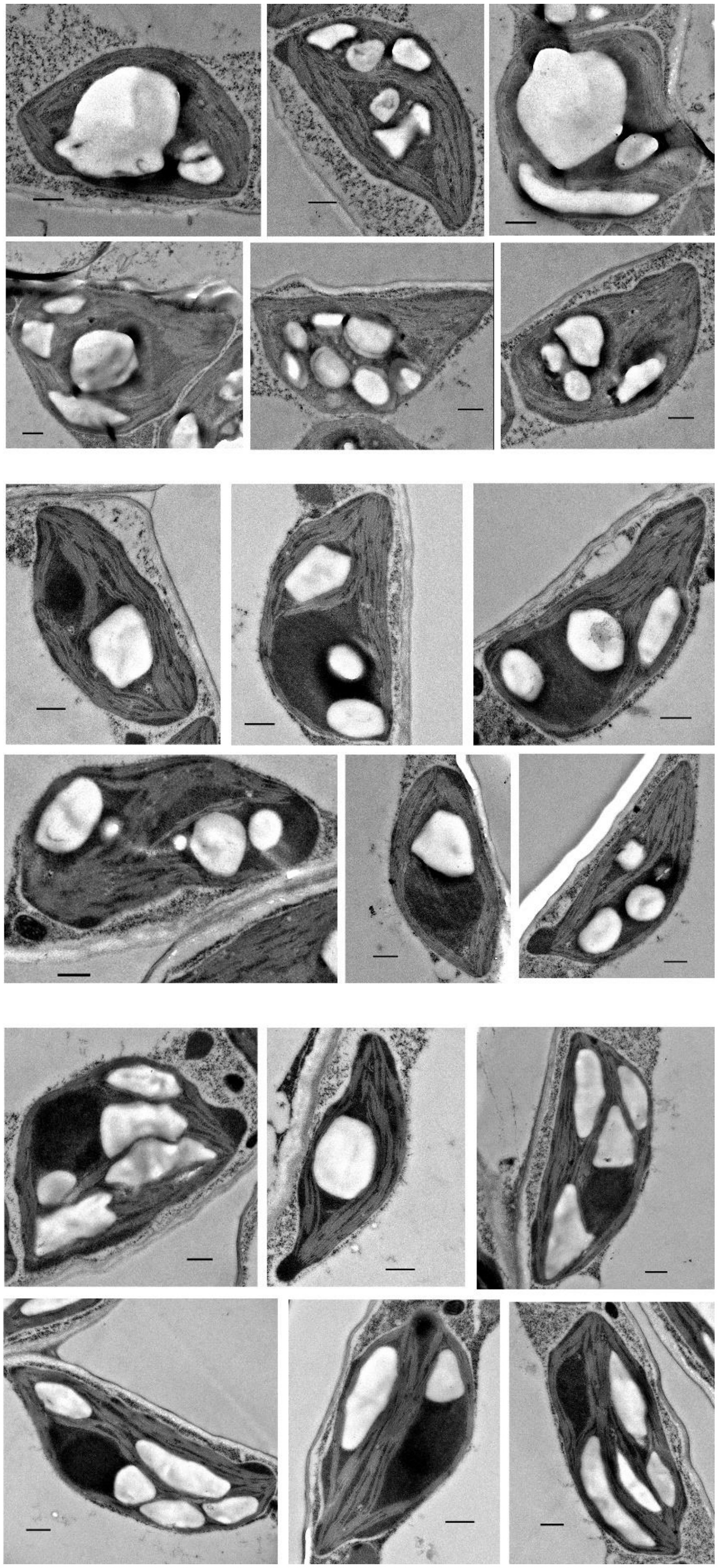

Figure S5. Additional representative TEM images for each of the three EP1S1S2 lines. See **Fig. 3** for transgene expression levels. Scale bar = 0.5 µm.

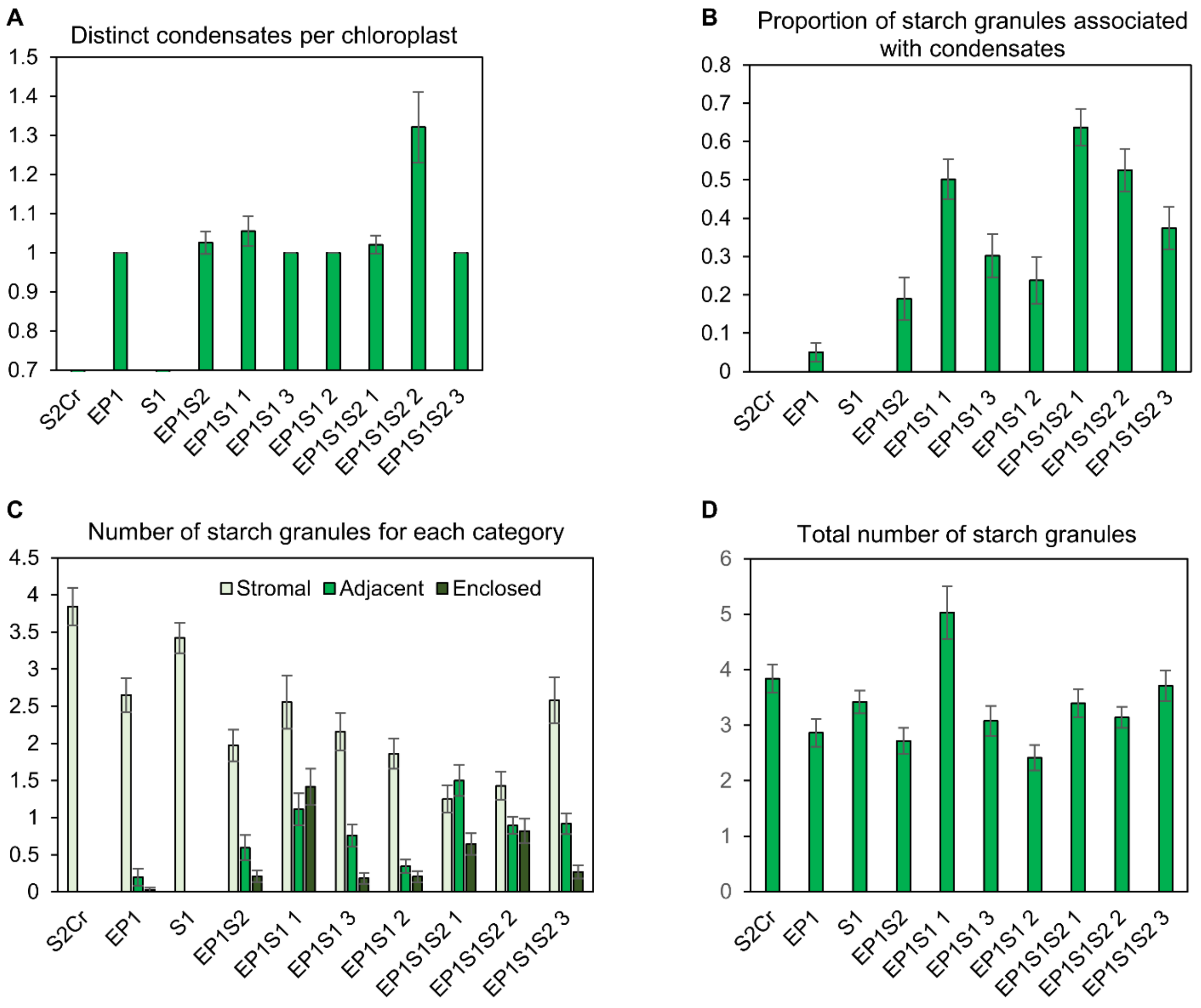

Figure S6. Additional starch granule parameters from area analysis of TEM images. A. The number of distinct condensates observed per chloroplast in each line. **B.** Proportion of total starch granules per chloroplast that are associated with the condensate (adjacent or enclosed granules) compared to those that were located in the stroma (stromal granules). **C.** Total number of starch granules per chloroplast classified as stromal, adjacent or enclosed. **D.** Total number of starch granules per chloroplast. Error bars show the mean ± SEM of 24-48 chloroplasts per line.

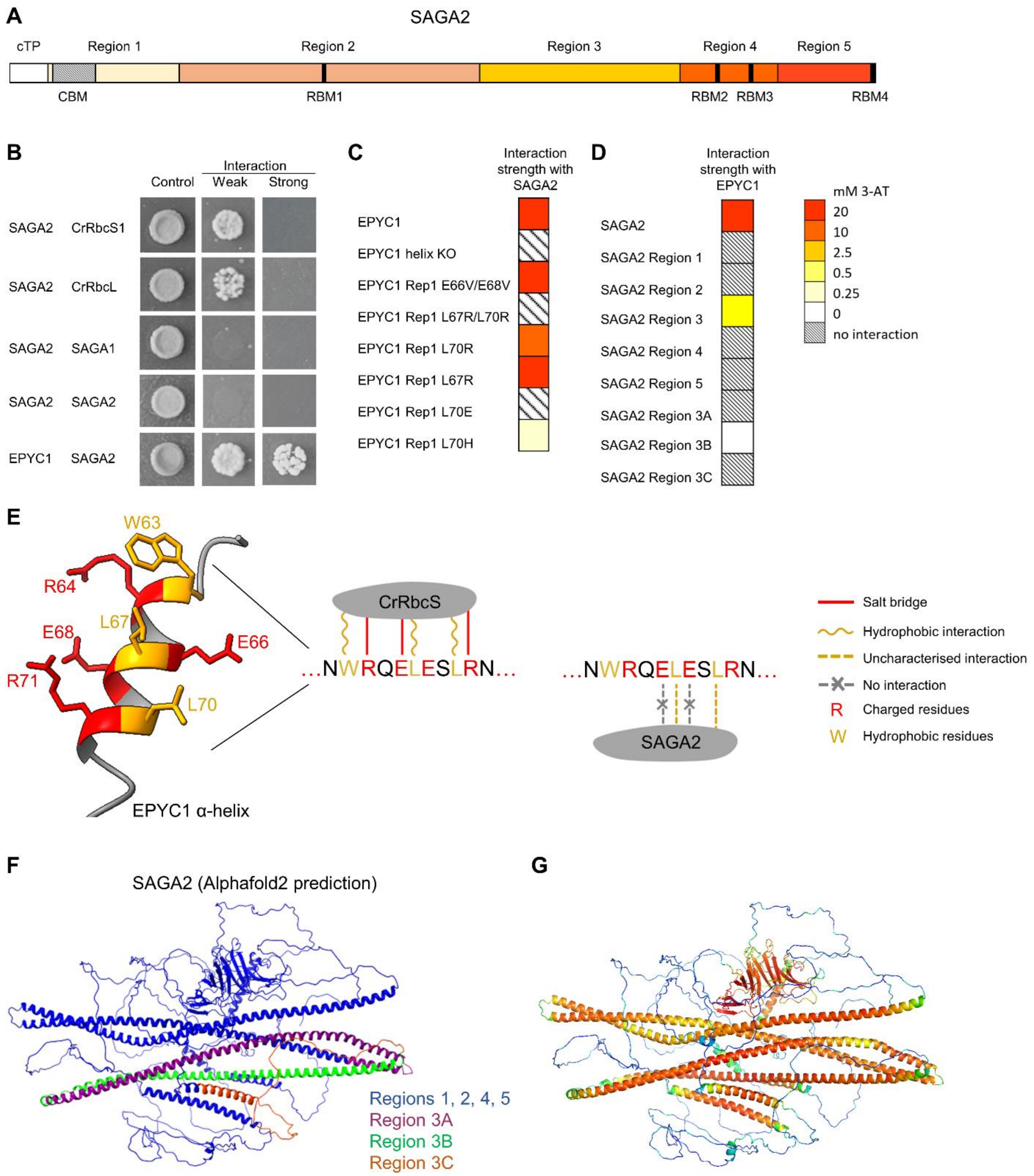

Figure S7. SAGA2 interacts with EPYC1 through hydrophobic residues on the EPYC1 α-helix. **A.** SAGA2 (Cre09.g394621, 1817 amino acid residues) contains a C-terminal carbohydrate binding motif (CBM) and four Rubisco binding motifs (RBMs, black bars). A predicted chloroplast transist peptide (cTP) and regions for interaction analysis (below) are shown. **B.** Yeast 2-hybrid (Y2H) assays supported protein-protein interactions between SAGA2 and the small subunit 1 of Rubisco in Chlamydomonas (CrRbcS1; Cre02.g120100), the large subunits of Rubisco (CrRbcL) and EPYC1. Control column shows

growth on CSM-L-W, whilst interaction columns show growth on CSM-L-W-H dropout media (weak), and with 10 mM 3-aminotriazole (3-AT) inhibitor (strong). **C.** Y2H assays showing the interaction strength between SAGA2 and EPYC1 with mutations in various residues of the α-helix. Interaction strength was scored by growth on increasing concentrations of 3-AT as in Atkinson et al. (26). The EPYC1 α-helix KO is a full length EPYC1 in which each of five α-helix sequences was replaced by alanine residues. In the subsequent samples just one repeat of EPYC1 is tested for interaction (Rep1, corresponding to amino acid residues 28–76 of the full-length protein), with one or more residues substituted. **D.** SAGA2 was split into 5 regions, with region 3 further subdivided into three parts (A-C), to test for interaction with EPYC1. **E.** Model of one of the repeat α-helix sequences of EPYC1 (residues 63-72) with hydrophobic residues shown in orange and charged residues shown in red. A comparison of the residues that interact with CrRbcS (19) and SAGA2 (from data in C) is shown. **F.** Predicted structure of SAGA2 (Alphafold2), with sections of region 3 highlighted in magenta (A), green (B) and orange (C), respectively. **G**. Predicted SAGA2 structure coloured by confidence score according to the Predicted Local Difference Distance Test (pLDDT), where red = high and blue = low. Please note that SAGA2 is predicted to contain several intrinsically disordered regions (IDRs), and the structural predictions of AlphaFold can be unreliable for IDRs.

Figure S8. SBF-SEM raw video data and 3-D reconstruction videos (see Movies S1-S12). Raw and 3-D reconstruction videos for the SAGA1 network in EP1S1 in **Fig. 4E** (‘pnas.2311013121.sm01.mp4’ and ‘pnas.2311013121.sm04.mp4’). Videos for stromal, enclosed and adjacent starch granules in EP1S1 in **Fig. 4F** (‘pnas.2311013121.sm02.mp4’ and ‘pnas.2311013121.sm05.mpg’). Videos for adjacent starch granules in EP1S1S2 in **Fig. 4G** (‘pnas.2311013121.sm03.mp4’ and ‘pnas.2311013121.sm06.mpg’). Videos for EP1-1 in **Fig. 1C** (‘pnas.2311013121.sm010.mp4’ and ‘pnas.2311013121.sm07.mp4’). Videos for S1-1 in **Fig. 1C** (‘pnas.2311013121.sm11.mp4’ and ‘pnas.2311013121.sm09.mp4’). Videos for $S2_{Cr}$ in **Fig. 1C** (‘pnas.2311013121.sm12.mp4’ and ‘pnas.2311013121.sm09.mp4’).

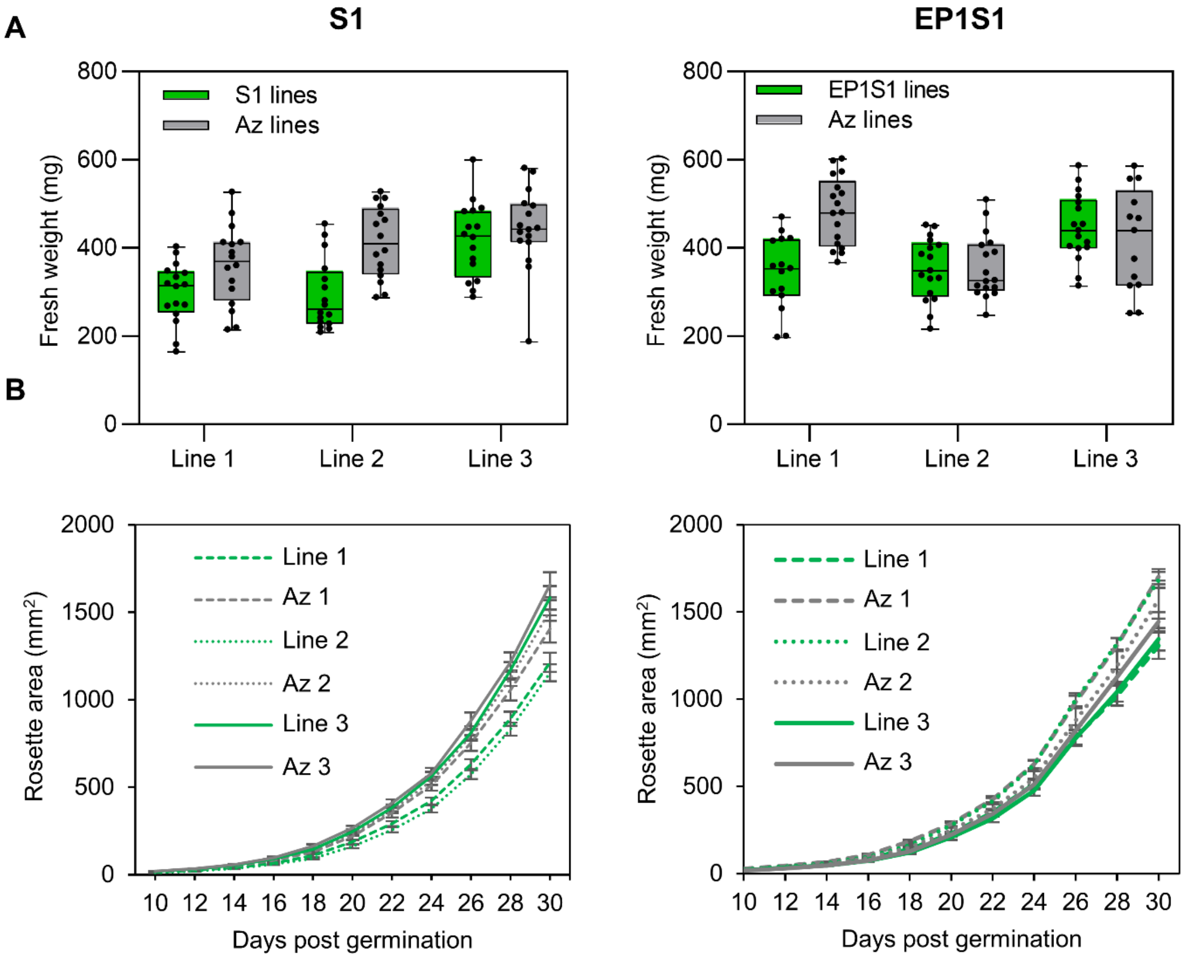

Figure S9. Fresh weight and rosette areas of S1 and EP1S1 lines. A. Fresh weight of S1 and EP1S1 lines and their azygous (Az) segregants 32 days after germination. **B.** Rosette expansion for S1 and EP1S1 lines measured over 30 days post germination. Error bars show the mean ± SEM of 12-21 individual rosettes.

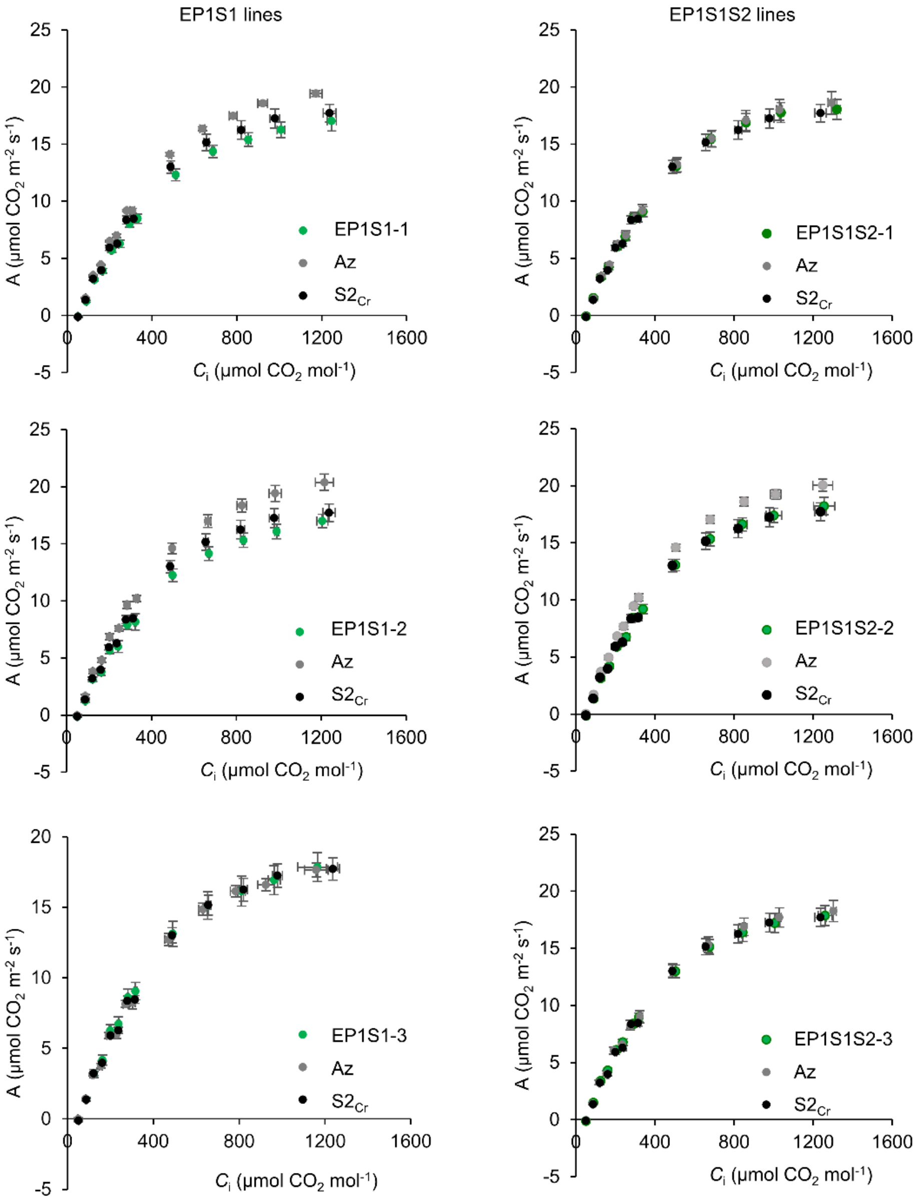

	EP1S1-1	Az1	EP1S1-2	Az2	EP1S1-3	Az3	$S2_{Cr}$
V_{cmax} (µmol CO_2 m^{-2} s^{-1})	44.0±2.7[ab]	46.1±2.8[ab]	52.1±2.0[a]	41.6±2.9[b]	46.7±2.1[ab]	44.2±2.5[ab]	45.2±1.9[ab]
J_{max} (µmol e^- m^{-2} s^{-1})	74.3±2.9[a]	81.7±3.9[a]	88.9±2.9[a]	73.6±4.1[a]	82.4±5.2[a]	78.3±2.2[a]	80.3±3.8[a]
Γ (µmol CO_2 m^{-2} s^{-1})	52.4±1.7[a]	50.6±2.1[a]	47.1±3.1[a]	48.1±2.8[a]	52.0±5[a]	45.0±3.4[a]	48.1±3.2[a]
G_s (mol H_2O m^{-2} s^{-1})	0.28±0.03[a]	0.22±0.03[a]	0.31±0.02[a]	0.22±0.03[a]	0.22±0.03[a]	0.22±0.03[a]	0.22±0.04[a]
G_m (mol CO_2 m^{-2} s^{-1})	0.042±0.003[a]	0.044±0.003[a]	0.048±0.001[a]	0.039±0.003[a]	0.045±0.003[a]	0.042±0.003[a]	0.041±0.002[a]
F_v/F_m	0.84±0.01[a]	0.84±0.01[a]	0.84±0.02[a]	0.84±0.01[a]	0.84±0.01[a]	0.84±0.02[a]	0.84±0.01[a]

	EP1S1S2-1	Az1	EP1S1S2-2	Az2	EP1S1S2-3	Az3	$S2_{Cr}$
V_{cmax} (µmol CO_2 m^{-2} s^{-1})	45.8±2.9[a]	47.7±2.9[a]	46.3±2.3[a]	50.7±2.0[a]	47.2±3.6[a]	47.5±4.5[a]	45.2±1.9[ab]
J_{max} (µmol e^- m^{-2} s^{-1})	80.2±4.0[a]	82.6±4.4[a]	81.3±3.2[a]	88.6±2.3[a]	79.3±4.1[a]	80.4±4.2[a]	80.3±3.8[a]
Γ (µmol CO_2 m^{-2} s^{-1})	46.0±3.1[a]	47.6±1.3[a]	51.8±1.9[a]	47.1±1.0[a]	49.1±0.9[a]	46.1±1.0[a]	48.1±3.2[a]
G_s (mol H_2O m^{-2} s^{-1})	0.33±0.04[a]	0.32±0.04[a]	0.32±0.04[a]	0.28±0.04[a]	0.25±0.05[a]	0.25±0.05[a]	0.22±0.04[a]
G_m (mol CO_2 m^{-2} s^{-1})	0.041±0.003[a]	0.040±0.002[a]	0.041±0.002[a]	0.049±.004[a]	0.048±0.006[a]	0.047±0.07[a]	0.041±0.002[a]
F_v/F_m	0.85±0.01[a]	0.85+0. 01[a]	0.85+0.01[a]	0.85±0.01[a]	0.85±0.01[a]	0.85±0.01[a]	0.84±0.01[a]

Figure S10. Net CO_2 assimilation (*A*) based on sub-stomatal [CO_2] (C_i) under saturating light (1500 µmol photons m^{-2} s^{-1}) for EP1S1 and EPS1S2 lines. Values show the mean ± SEM of 5-8

individual measurements on separate rosettes for EP1S1 (left) and EP1S1S2 (right) plants, their azygous segregants and the background line $S2_{Cr}$. Variables derived from gas exchange data include maximum rate of Rubisco carboxylation (V_{cmax}), maximum electron transport rate (J_{max}), CO_2 compensation point (Γ) , stomatal conductance (G_s), mesophyll conductance (G_m) and the maximum quantum yield of photosystem II (F_v/F_m). Letters indicate significant difference ($p < 0.05$) as determined by one-way ANOVA followed by Tukey's honestly significant difference (HSD) post-hoc tests.

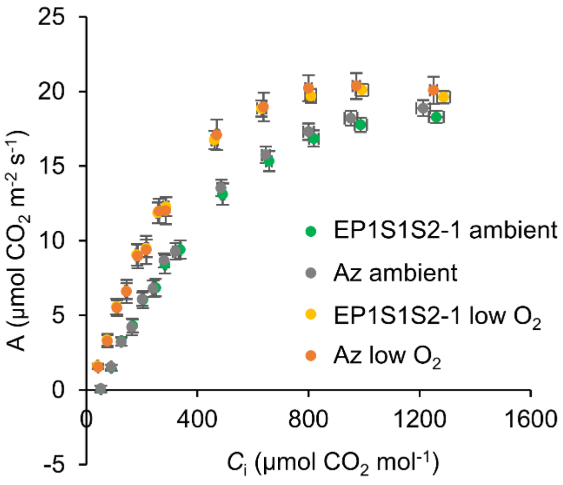

	Ambient		Low O_2	
	**EP1S1S2-1**	**Az1**	**EP1S1S2-1**	**Az1**
V_{cmax} (µmol CO_2 m^{-2} s^{-1})	46.8±3.4[a]	48.4±4.4[a]	42.9±2.1[a]	43.6±2.8[a]
J_{max} (µmol e^- m^{-2} s^{-1})	83.1±4.1[a]	85.9±4.8[a]	85.1±3.2[a]	88.9±5.1[a]
Γ (µmol CO_2 m^{-2} s^{-1})	46.9±1.1[a]	46.8±2.2[a]	11.4±3.2[b]	9.0±1.2[b]
A_{400} (µmol CO_2 m^{-2} s^{-1})	9.4±0.4[a]	9.3±.0.7[a]	12.3±0.8[b]	12.1±0.7[b]
Initial slope (*A*/*Ci*)	35.3±1.9[a]	36.8±.5.7[a]	46.0±2.2[b]	47.2±2.8[b]
G_s (mol H_2O m^{-2} s^{-1})	0.35±0.02[a]	0.30±0.06[a]	0.23±0.03[a]	0.35±0.02[a]
G_m (mol CO_2 m^{-2} s^{-1})	0.041±0.002[a]	0.042±0.009[ab]	0.054±0.004[ab]	0.055±0.004[b]

Figure S11. Net CO_2 assimilation (*A*) based on sub-stomatal [CO_2] (C_i) under saturating light (1500 µmol photons m^{-2} s^{-1}) for EPS1S2 under photorespiratory and non-photorespiratory conditions. Values show the mean ± SEM of 6 individual measurements on separate rosettes for EP1S1S2-1 plants and azygous segregants measured under ambient O_2 and 2% O_2. Variables derived from gas exchange data include maximum rate of Rubisco carboxylation (V_{cmax}), maximum electron transport rate (J_{max}), CO_2 compensation point (Γ), CO_2 assimilation rate at near ambient CO_2 (A_{400}), carboxylation efficiency taken as the initial slope between 50 and 350 µmol mol^{-1} ambient CO_2, stomatal conductance (G_s) and mesophyll conductance (G_m). Letters indicate significant difference ($p < 0.05$) as determined by one-way ANOVA followed by Tukey's honestly significant difference (HSD) post-hoc tests.

Figure S12. Sequence maps of plasmid vector used in this study (see Datasets S3-S8). See **Table S1/Dataset S1** for a summary list of vectors.

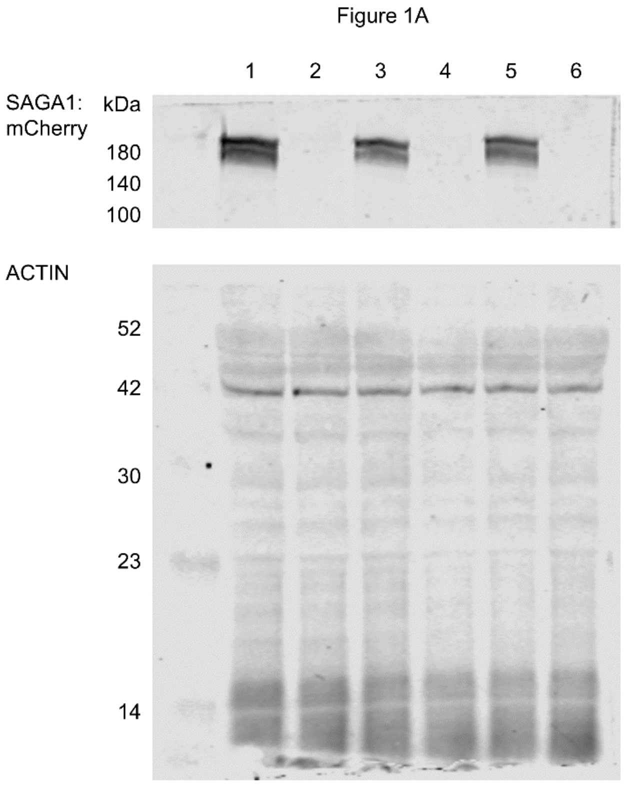

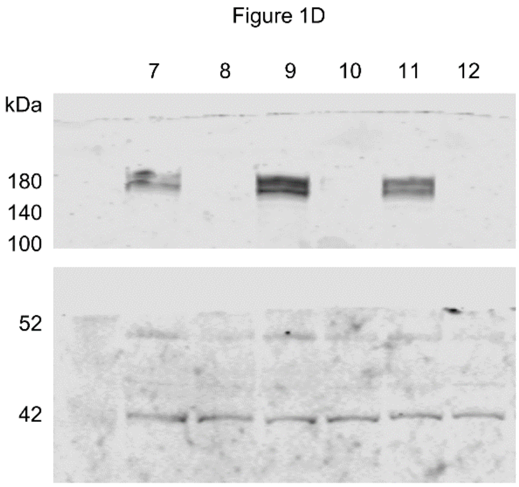

Lanes:
1. S1_1
2. S1_1 AZ
3. S1_2
4. S1_2 AZ
5. S1_3
6. S1_3 AZ
7. EP1S1_1
8. EP1S1_1 AZ
9. EP1S1_2
10. EP1S1_2 AZ
11. EP1S1_3
12. EP1S1_3 AZ

Figure 2A

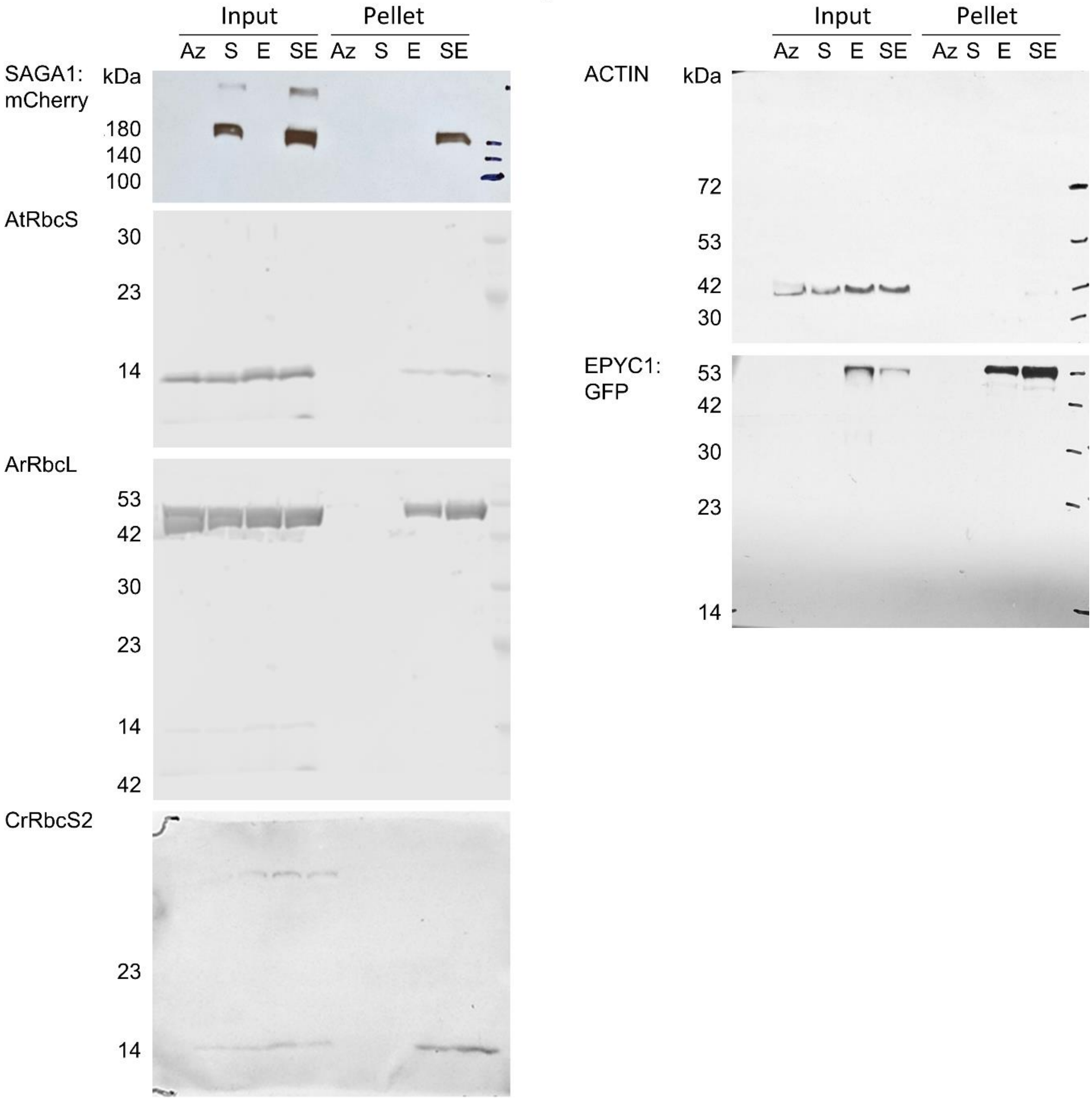
Input
Pellet
Az S E SE Az S E SE
SAGA1: mCherry
kDa
180
140
100
AtRbcS
30
23
14
ArRbcL
53
42
30
23
14
42
CrRbcS2
23
14
ACTIN
72
53
42
30
EPYC1: GFP
53
42
30
23
14

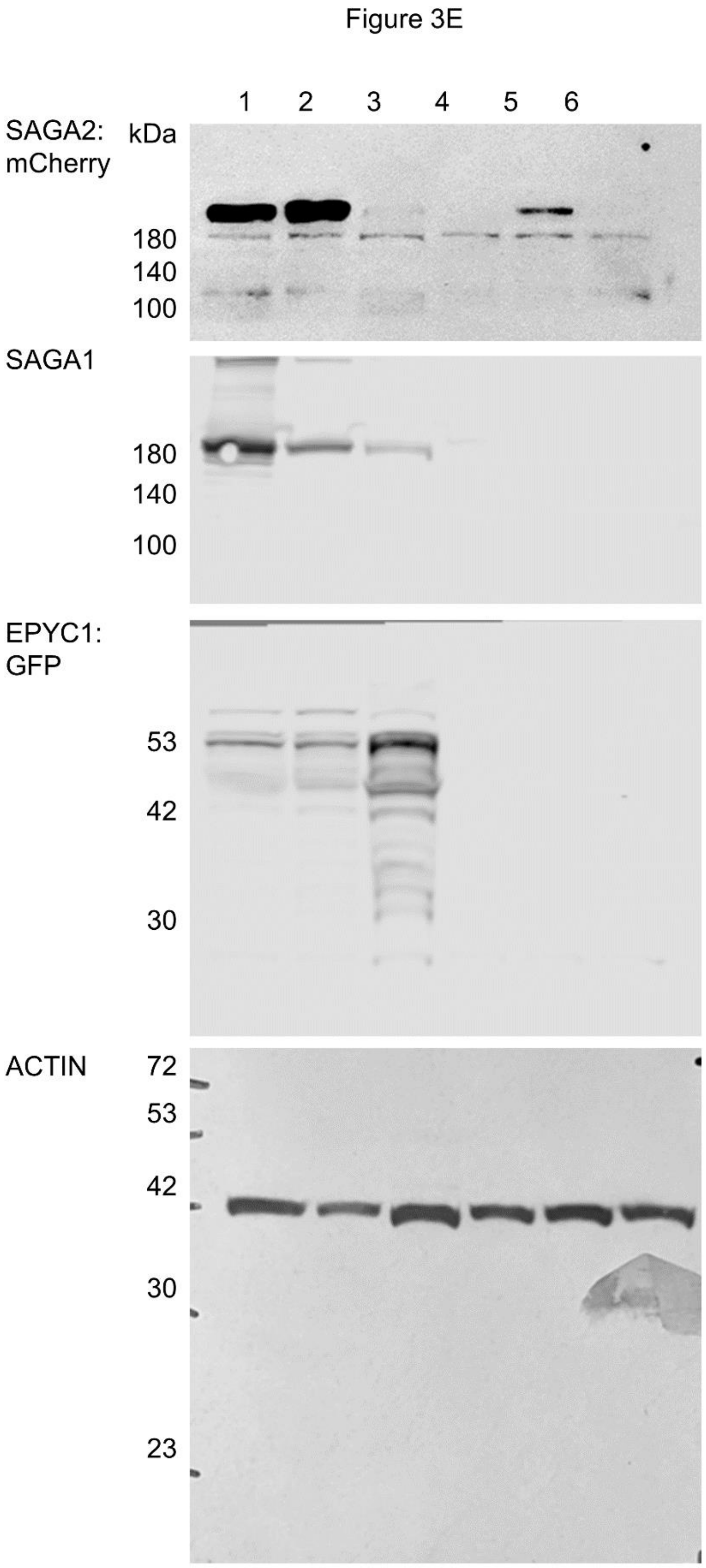

Lanes:
1. EPS1S2_1
2. EPS1S2_2
3. EPS1S2_3
4. EPS1S2_1 AZ
5. EPS1S2_2 AZ
6. EPS1S2_3 AZ

Figure S13. Uncropped immunoblot images from main figures.

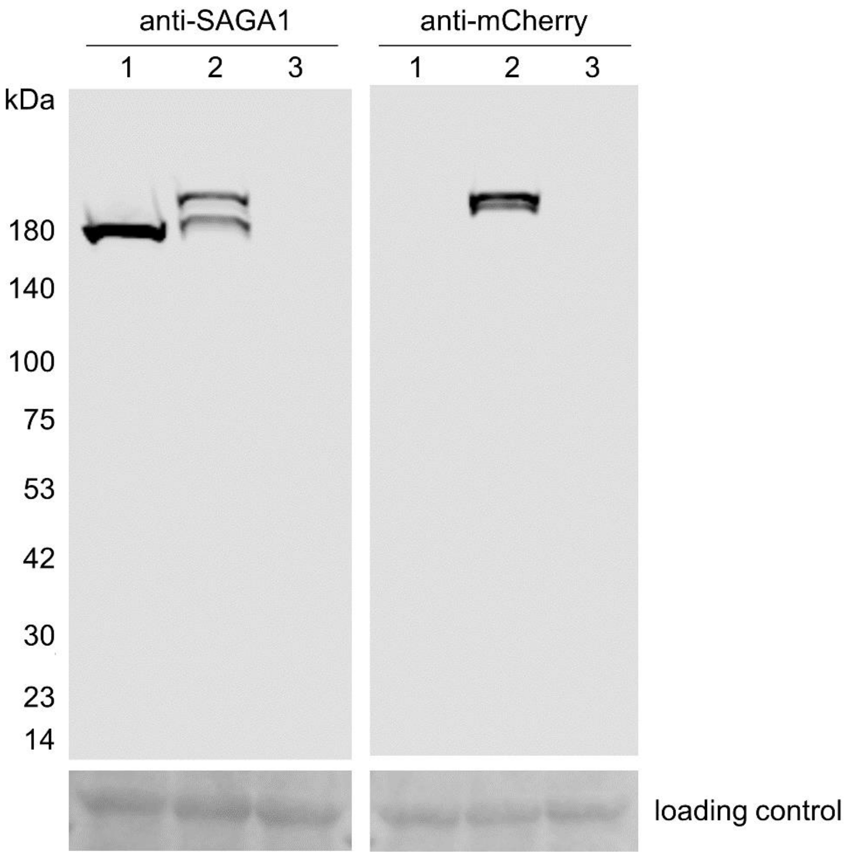

Lanes:
1. SAGA1, EPYC1:GFP
2. SAGA1:mCherry, EPYC1:GFP
3. EPYC1:GFP

Figure S14. Immunoblots showing expression of SAGA1 and SAGA1:mCherry. Protein extracts from plants expression untagged SAGA1 and EPYC1:GFP (lane 1), SAGA1:mCherry and EPYC1:GFP (EP1S1, lane 2) were probed with either anti-SAGA1 or anti-mCherry antibodies. Plants expressing only EPYC1:GFP (lane 3, EP1) were included as a control. The higher bands in lane 2 correspond to SAGA1:mCherry, the lower bands correspond to untagged SAGA1. The presence of two bands in lane 2 for anti-SAGA1 indicates that mCherry is prone to cleavage (as seen in Fig. 1). The absence of free mCherry (27 kDa) indicates that cleaved mCherry is rapidly degraded.

Table S1. List of plasmid vectors used in this study (**see Dataset S1**). See **Fig. S12** for vector maps as .gb files (**Datasets S3-S8**).

Table S2. SBF-SEM staining protocol.

Steps	Solution	Time	Temperature	Microwave watt	Vacuum
1	1.5% Glutaraldehyde<br>2.5% Paraformaldehyde<br>5% Sucrose in 0.1 M NaCac	2 x 14 min<br>(2min on/off cycles)	Room temperature	100	On
2	0.1 M NaCac	1 immediate.<br>Then 2 x 40 s.		100	On
3	2% OsO_4 in 0.1 M NaCac	2 x 14 min<br>(2min on/off cycles)		100	On
4	2.5% $K_4[Fe(CN)_6] \cdot 3H_2O$<br>in 0.1 M NaCac	2 x 14 min<br>(2 min on/off cycles)		100	On
5	Water wash	1 immediate.<br>Then 2 x 40 s.		100	On
6	1% thiocarbohydrazide unbuffered	2 x 14 min<br>(2 min on/off cycles)		100	On
7	Water wash	1 immediate.<br>Then 2 x 40 s.		100	On
8	2% OsO_4 aqueous	2 x 14 minutes<br>(2 min on/off cycles)		100	On
9	Water wash	1 immediate.<br>Then 2 x 40 s.		100	On
10	Dehydration series in ethanol (25%, 50%, 75%, 3 x 100%)	40 s each		250	Off
11	Infiltration series in Durcupan:ethanol mixes (25%, 50%, 75%, 90%, 2 x 100%)	3 min each		150	On
12	100% Durcupan	overnight		N/A	N/A
13	Polymerisation	30 min	60°C	250	Off
14	Polymerisation	90 min	100°C	450	Off

Table S3. Gene coding sequences used for Yeast Two-Hybrid assays (see Dataset S2).
